# Metabolic and morphological profile in skeletal muscle of healthy boys and girls

**DOI:** 10.14814/phy2.15414

**Published:** 2022-08-19

**Authors:** Mona Esbjörnsson, Barbara Norman, Monica Dahlström, Jan Gierup, Eva Jansson

**Affiliations:** ^1^ Division of Clinical Physiology, Department of Laboratory Medicine Karolinska Institutet Stockholm Sweden; ^2^ Unit of Clinical Physiology Karolinska University Hospital Stockholm Sweden; ^3^ Sollentuna Sweden; ^4^ Lidingö Sweden

**Keywords:** age, children, cross‐sectional area, fiber types, metabolic enzymes, sex

## Abstract

It was hypothesized that the typical adult pattern of higher glycolytic capacity in skeletal muscle of males compared to females is not observed in children and that fiber cross‐sectional area (CSA) is a determinant of glycolytic capacity in children. Biopsies were performed in vastus lateralis in 9–12 years‐old healthy boys and girls (*N* = 27). Fiber types were classified by myofibrillar ATPase staining and CSA was measured using planimetry. Citrate synthase (CS) and lactate dehydrogenase (LD) were analyzed using fluorometric and spectrophotometric methods. There was no significant difference between boys and girls in CS activity (0.45 ± 0.1 μkat g^−1^ dry muscle in boys and 0.42 ± 0.1 in girls) or LD activity (24 ± 6 μkat g^−1^ dry muscle in boys and 25 ± 7 in girls). CSA did not differ between boys and girls. CS was inversely related to type I CSA (*r* = −0.62, *p* < 0.001) and LD was directly related to type IIA (*r* = 0.63, *p* < 0.001) and type IIB CSA (*r* = 0.72, *p* < 0.001). CSA was a significant determinant of CS and LD, even after adjusting for sex and relative fiber type area in multiple regression analysis. This suggests that the typical adult pattern of higher muscle glycolytic capacity in males than in females, as estimated by LD activity, was not observed in children. Sex‐specific patterns in glycolytic capacity thus appear to develop during the transition from childhood to adulthood. In addition, fiber CSA was a strong determinant of both muscle glycolytic and oxidative capacity in children, regardless of sex.

## Graphical abstract

No sex difference was found in skeletal muscle lactate dehydrogenase (LD) activity in 10 years old healthy children. This suggests that the typical adult pattern of higher muscle glycolytic capacity in males than in females develops during the transition from childhood to adulthood. In addition, muscle fiber cross‐sectional area (CSA) was a statistical determinant of muscle LD activity in the children, supporting old data showing that animal size was a determinant of muscle LD activity.

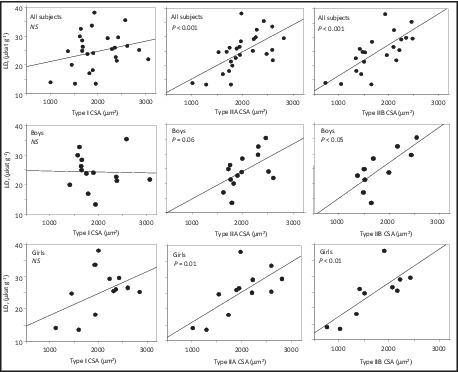

## 1 INTRODUCTION

ATP in skeletal muscle is produced through oxidative metabolism, but also by anerobic glycolysis, especially during limited oxygen supply or high‐power output. Metabolic enzymes are often analyzed in vitro in skeletal muscle to describe the capacity of oxidative and glycolytic ATP production pathways. In studies of adult human skeletal muscle, higher activity of glycolytic enzymes in males than in females is generally evident (Esbjornsson et al., 1993; Essen‐Gustavsson & Borges, 1986; Gauthier et al., 1992; Green et al., 1984; Jaworowski et al., 2002; Komi & Karlsson, 1978; Simoneau et al., 1985; Simoneau & Bouchard, 1989). However, activity of oxidative enzymes are generally not found to be higher in males than females (Esbjornsson et al., 1993; Essen‐Gustavsson & Borges, 1986; Gauthier et al., 1992; Green et al., 1984; Jaworowski et al., 2002; Simoneau et al., 1985; Simoneau & Bouchard, 1989). Early data from Eriksson et al. (1973), which demonstrated lower activity of glycolytic enzymes in vastus lateralis in 11–13 years old boys than in adult males, suggested that a sex difference in glycolytic enzymes develops during puberty (Eriksson et al., 1973). Higher glycolytic enzyme activity in adult males compared with boys has also been reported in a non‐locomotor muscle (Kaczor et al., 2005). Berg et al. (1986) analyzed glycolytic enzymes in both boys and girls, but data was not analyzed by sex (Berg et al., 1986). To the best of our knowledge, there are thus currently no data directly comparing boys and girls. Increased knowledge about the capacity of anaerobic glycolysis in skeletal muscle in boys and girls may add to the understanding of physical performance in children, and to changes during transition from child‐to‐adult.

The first aim of the present study was therefore to compare metabolic profile in skeletal muscle of boys and girls by analyzing selected metabolic enzymes. It was hypothesized that the typical adult pattern of higher glycolytic capacity in males than females would not be observed in skeletal muscle in children.

A second aim was to investigate the relationship between glycolytic enzyme activity and muscle fiber cross‐sectional areas. A previous study compared adult males and females in terms of metabolic enzyme activities in skeletal muscle and reported that the cross‐sectional area was a strong determinant of glycolytic enzyme activity (Jaworowski et al., 2002). However, it cannot be excluded that the relationship between glycolytic enzymes and cross‐sectional areas in adult muscle is influenced by sex hormones. Therefore, we also tested the hypothesis that fiber size is even a determinant of glycolytic enzyme activity in children, where the influence of sex hormones is known to be less than in adults.

## 2 METHODS

### 2.1 Participants

To increase the statistical power of the study we pooled data from two cohorts of children. In total, 27 healthy children of both sexes (15 boys and 12 girls) were included in the study (Table 1). Twelve were recruited from children who had recently started professional dance training and are referred to as ‘novice‐dancers’. Fifteen of them were recruited from children who had undergone minor surgery (e.g., inguinal hernia) but who were otherwise healthy and not prevented from being physically active and are referred to as ‘non‐dancers’. Body weight and height were measured and Body Mass Index (BMI) was calculated. The children were 10 (range 9–12) years old. All girls were pre menarche as reported by the parents. Non‐dancers were taller and heavier than novice‐dancers and on average 6 months older, but no sex differences or interaction terms between subject group and sex were identified in a two‐way ANOVA. BMI did not differ between the group or sex (Table 1).

**TABLE 1:** Anthropometrics, muscle fiber characteristics and enzyme activities in boys and girls. Data for BMI and all muscle outcomes are presented as pooled data of the two children cohorts—novice‐dancers and non‐dancers—as no significant effects of group or interactions between sex and group were found. Data divided by group and sex are also presented

Sex	Boys	Girls	*p*‐values			Boys		Girls	
Group	Both groups	Both groups				Novice‐dancers	Non‐dancers	Novice‐dancers	Non‐dancers
	*N* = 15	*N* = 12	Sex (S)	Group (G)	S × G	*N* = 5	*N* = 10	*N* = 7	*N* = 5
Age (years)			NS	<0.05	NS	10 ± 0	10 ± 1	10 ± 1	11 ± 1
Body weight (kg)			NS	<0.01	NS	28 ± 3	37 ± 8	29 ± 2	35 ± 7
Height (cm)			NS	<0.01	NS	134 ± 6	144 ± 9	138 ± 5	148 ± 7
BMI (kg m^−2^)	17 ± 2	16 ± 2	NS	NS	NS	15 ± 0	18 ± 2	16 ± 1	16 ± 2
Type I CSA (μm^2^)	2013 ± 411	2065 ± 503	NS	NS	NS	1773 ± 213	2133 ± 441	1987 ± 578	2173 ± 411
Type IIA CSA (μm^2^)	1998 ± 322	1985 ± 543	NS	NS	NS	1900 ± 220	2048 ± 363	1920 ± 516	2076 ± 628
Type IIB CSA (μm^2^)	1796^a^ ± 355	1681^a^ ± 555	NS	NS	NS	1986^a^ ± 280	1732^a^ ± 368	1557^a^ ± 551	1805^a^ ± 592
Type IIA/I CSA	1.0 ± 0.2	1.0 ± 0.2	NS	NS	NS	1.1 ± 0.2	1.0 ± 0.2	1.0 ± 0.2	0.9 ± 0.2
Type I (%)	64 ± 8	61 ± 9	NS	NS	NS	67 ± 11	63 ± 6	63 ± 8	57 ± 10
Type IIA (%)	28 ± 5	30 ± 7	NS	NS	NS	27 ± 6	28 ± 5	30 ± 7	29 ± 6
Type IIB (%)	7 ± 6	11 ± 6	NS	=0.05	NS	6 ± 8	9 ± 5	6 ± 6	13 ± 6
Type IIC (%)	1.0 ± 0.1	0.1 ± 0.3	NS	NS	NS	0.5 ± 1	1 ± 2	0.1 ± 0.1	0.2 ± 0.4
Type I CSA (%)	65 ± 7	63 ± 8	NS	NS	NS	66 ± 8	65 ± 7	65 ± 6	61 ± 10
Type IIA CSA (%)	28 ± 6	30 ± 7	NS	NS	NS	28 ± 4	28 ± 7	30 ± 7	29 ± 7
Type IIB CSA (%)	7 ± 6	7 ± 6	NS	NS	NS	6 ± 7	7 ± 5	5 ± 5	9 ± 8
Citrate synthase (CS), (μkat g^−1^ dry muscle)	0.45 ± 0.1	0.42 ± 0.1	NS	NS	NS	0.45 ± 0.0	0.45 ± 0.1	0.45 ± 0.1	0.37 ± 0.0
Lactate dehydrogenase (LD), (μkat g^−1^ dry muscle)	24 ± 6	25 ± 7	NS	NS	NS	25 ± 4	24 ± 7	27 ± 8	23 ± 7
LD/CS	56 ± 17	63 ± 23	NS	NS	NS	56 ± 7	56 ± 20	64 ± 23	63 ± 7
*N* ^a^	12	10				3	9	5	5

*Note*: Values are mean and SD.

Abbreviations: BMI, body mass index; CSA, cross‐sectional area; *N*, number of subjects; type I CSA (%), relative type I fiber area.

^a^ Indicate number of individulas of the calculated mean value of type IIB CSA.

Regardless of group, all children had been physically active on average 2 (range 0–7) hours per week in various types of activities such as basketball, ice hockey, riding, soccer or swimming, for 1–6 years in addition to their regular physical education at school. Three of the children in the group of novice‐dancers had also been dancing 1–2 times per week for a few years. The novice‐dancers started their education at the Swedish Ballet School by mainly slow method training a few weeks before the start of the present study.

Data of muscle fiber type composition and CSA have been previously published (Dahlstrom et al., 1997; Esbjornsson et al., 2021). The present investigation extends the previously published data by elucidating correlations to metabolic enzymes. All children and their parents were fully informed verbally and in writing before the children consented to participate in the study with the parent's approval. The study was approved by the Ethics Committee of the Karolinska Hospital (88:188) and of the St Göran's Hospital (90:80).

### 2.2 Experimental protocol and analyses

Percutaneous needle biopsies (Bergstrom, 1975) were performed in *vastus lateralis* from the non‐dancers under general anesthesia when undergoing minor surgery and from novice‐dancers under local anesthesia. The biopsies were mounted in an embedding medium, frozen in isopentane precooled with liquid nitrogen and stored at −80°C until analysis. The biopsies were carefully oriented and freeze‐cross‐sectioned with a thickness of 10 μm and analyzed histochemically for the fiber types I, IIA, IIB and IIC using a myofibrillar adenosine triphosphatase (ATPase) staining (Schantz et al., 1982). The cross‐sectional area (CSA) of the various fiber types was measured by planimetry from a NADH‐dehydrogenase staining (Novikoff et al., 1961) as described earlier (Esbjornsson et al., 2021). Cross‐cut sections were freeze‐dried, weighed (50–100 μg), and homogenized in ice‐cooled 0.1 M phosphate buffer, pH 7.7 with 0.5% BSA (1 μg dry muscle per μl buffer). Citrate synthase (CS) activity was determined by a fluorometric method at 25°C according to the principles of Lowry and Passonneau (1972) and 10 μl homogenate was added to 1 ml reagent solution containing 0.1 M Tris buffer pH 8.0, 2.5 mM EDTA, 0.5 mM l‐malate, 8 pg/ml malate dehydrogenase (MDH), and 0.005 mM NAD. The reaction was started with 5 μl 12 mM acetyl‐CoA; 0.3 mM NADH was used as a standard (Lin et al., 1988). Total lactate dehydrogenase (LD) activity was determined by a spectrophotometric method at 37°C as recommended by the Scandianavian Enzyme Committee (Keiding et al., 1974) and 4 μl homogenate was added to 1 ml reagent solution containing 56 mM Tris buffer pH 7.4, 5.6 mM EDTA, 0.17 mM NADH. The reaction was started with 100 μl 14 mM pyruvate. Enzyme activities were expressed as μkat per g dry muscle. For ethical reasons and because of the children's thin muscle bellies all measurements were based on a single biopsy sample per child. The coefficient of variation for area measurements was previously reported to be 10%–15% when measurements were performed from a single biopsy in a similar manner as in the present study (Blomstrand et al., 1984).

### 2.3 Calculations

Fiber type composition was expressed as the relative number of the different fiber types; I%, IIA%, IIB% and type IIC%. Fiber type composition was also expressed as the relative fiber type area for type I, according to the formula: (type I% + IIC%) × type I CSA × 100 ((type I% + IIC%) × type I CSA + type IIA% × type IIA CSA + type IIB% × type IIB CSA)^−1^. The relative type IIA and type IIB were calculated using the corresponding equations.

### 2.4 Statistics

A two‐way ANOVA was applied to analyze the effects of sex (boys and girls) and subject group (novice‐dancers or non‐dancers) on background characteristics, fiber type variables and the enzyme activities, LD, CS and LD/CS (Table 1). Pearson's single linear regression analysis was performed between enzyme activities (*y* = LD, CS or LD/CS) and CSAs (*x* = type I, IIA or IIB). Multiple regression analysis was applied to analyze the influence of CSAs, sex, subject group, and relative type I fiber area (independent variables) on enzyme activity (dependent variable). Differences and correlations were accepted as statistically significant at the level of *p* < 0.05.

## 3 RESULTS

### 3.1 Participants

The purpose of the study was to compare boys and girls. Therefore, pooled data for each sex from the two children cohorts (group) were presented as the main outcome of the muscle variables, even though group was included in the two‐way ANOVAs and the multiple correlation analyses, see Methods. The pooling of data from the two cohorts was justified by the lack of main effects of group or interactions between sex and group in the ANOVAs for any of the skeletal muscle outcomes (Table 1).

### 3.2 Fiber type characteristics

**FIGURE 1:**
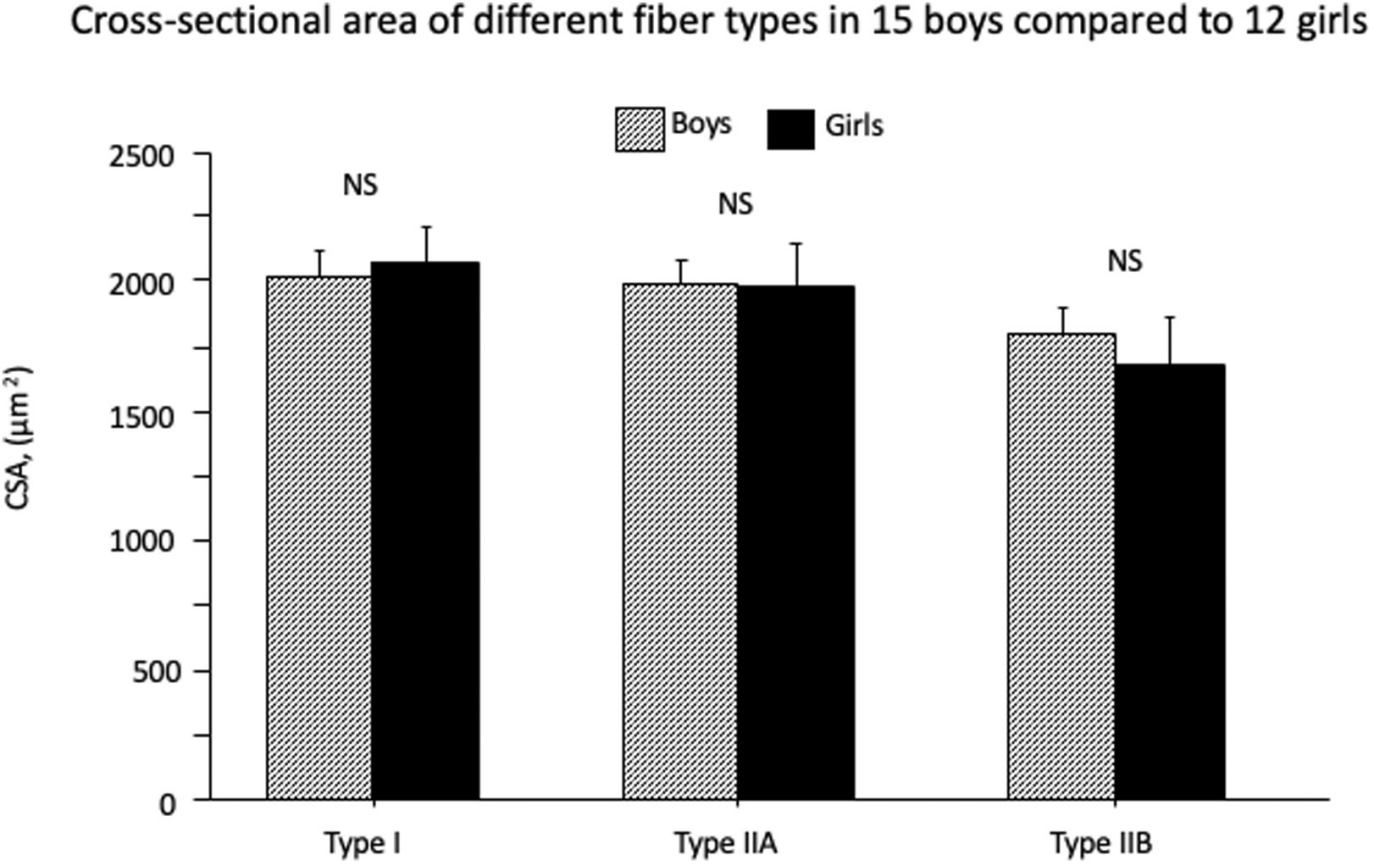
Muscle fiber cross‐sectional area (CSA) of type I, IIA and IIB in 15 boys and 12 girls. Values are mean and SE. NS = non‐significant.

There were no significant differences in fiber CSA between boys and girls for any of the fiber types (Table 1; Figure 1).

### 3.3 Enzymes

**FIGURE 2:**
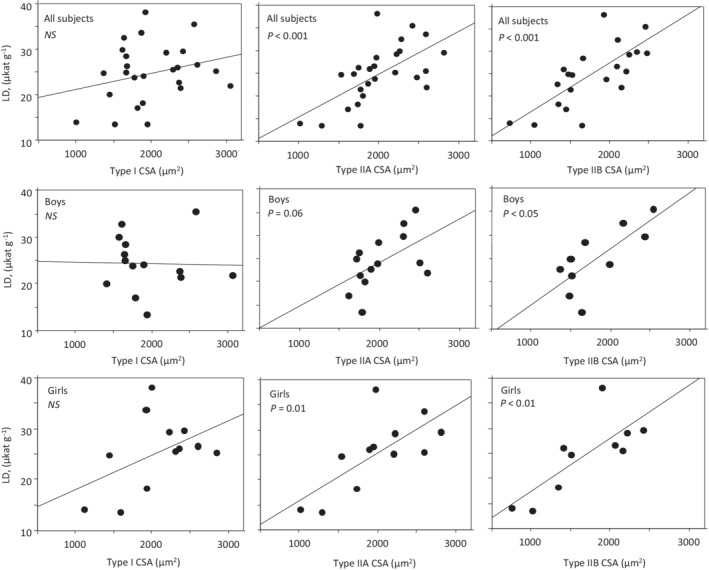
Relationship between lactate dehydrogenase (LD) activity and muscle fiber cross‐sectional area (CSA) of type I, IIA and IIB in all subjects and in boys and girls. The lines are the regression lines and *p*‐values are given.

**FIGURE 3:**
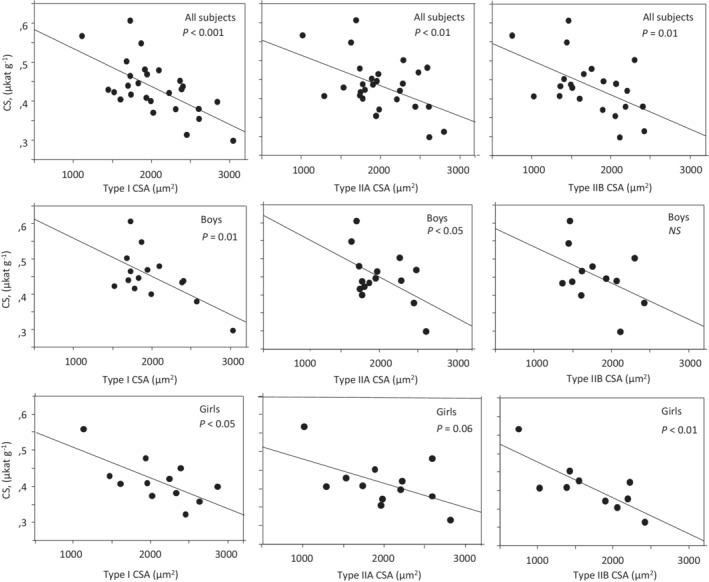
Relationship between citrate synthase (CS) activity and muscle fiber cross‐sectional area (CSA) of type I, IIA and IIB in all subjects and in boys and girls. The line is the regression line and *p*‐values are given.

**FIGURE 4:**
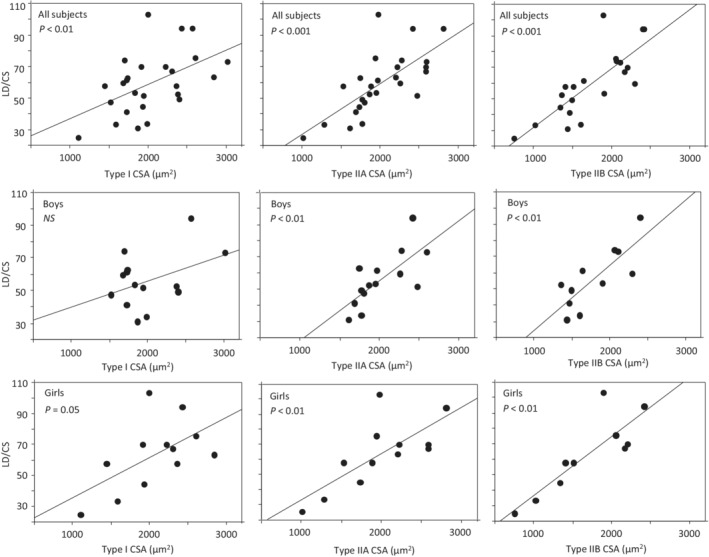
Relationship between LD/CS activity and muscle fiber cross‐sectional area (CSA) of type I, IIA and IIB in all subjects and in boys and girls. The line is the regression line and *p*‐values are given.

There was no significant difference in CS or LD activity between boys and girls (Table 1). Correlations were found between LD, CS activity and the LD to CS ratio (LD/CS) on the one hand and fiber CSA on the other. The CS activity was negatively correlated with fiber CSA, whereas LD activity and LD to CS ratio (LD/CS) were positively correlated to fiber CSA (Figures 2, 3, 4). For CS the correlation was somewhat stronger to type I CSA (*r* = −0.62, *p* < 0.001) than to type IIA CSA (*r* = −0.49, *p* < 0.01) and type IIB (*r* = −0.53, *p* < 0.01). The opposite was apparent for LD, with stronger correlations to type IIA (*r* = 0.63, *p* < 0.001) and type IIB CSA (*r* = 0.72, *p* < 0.001) than to type I CSA (*r* = 0.25, *p* < 0.22). Generally, the same pattern was observed regardless of whether subjects were pooled together or divided into boys and girls (Figures 2, 3, 4). Multiple correlations further supported that CSA was a determinant of CS, LD and LD/CS after adjustment for sex, subject group and relative type I fiber area (Table 2).

**TABLE 2:** Multiple regression analysis with enzyme activity as dependent variable and fiber CSA, sex, group (novice‐dancer or non‐dancers) and relative type I fiber area (%) as independent variables

Enzyme	*p* <							
	Fiber CSA, μm^2^		Sex	Group	Type I CSA (%)	Std coeff	*R* ^2^	N
CS	Type I	0.002	NS	NS	NS	−0.58	0.45	27
LD	Type IIA	0.001	NS	0.03	NS	0.62	0.54	26
LD	Type IIB	0.001	NS	NS	NS	0.68	0.60	21
LD/CS	Type IIA	0.001	NS	NS	NS	0.72	0.56	26
LD/CS	Type IIB	0.001	NS	NS	NS	0.86	0.70	21

*Note*: Units are μkat g^−1^ dry muscle.

Abbreviations: CS, citrate synthase; CSA, cross‐sectional area; LD, lactate dehydrogenase; *N*, number of subjects; *R* ^2^, coefficient of determination; std coeff, standardized coefficient; type I CSA (%), relative type I fiber area.

## 4 DISCUSSION

### 4.1 Major findings—first hypothesis

To the best of our knowledge, this is the first study of metabolic enzymes in skeletal muscle of healthy children of both sexes. The first hypothesis that the typical adult pattern of higher glycolytic capacity in males than in females would not be observed in children, was confirmed. These results are consistent with our previously reported data regarding skeletal muscle morphology in boys and girls, where the higher ratio between fiber CSA of the glycolytic type II fibers and oxidative type I fibers in adult males compared to females was not observed in children (Esbjornsson et al., 2021).

The reason for the development of higher glycolytic capacity in males than in females during the transition from child‐to‐adult is unknown and was beyond the scope of the current study and could only be speculated upon. In the present study, the CSA was a strong determinant of LD in children. The larger CSA in adult males than females could therefore contribute to explain the sex‐specific LD enzyme pattern in adults. However, other factors than the CSA in adults could also contribute to explain individual differences in LD activity. Firstly, increase in testosterone levels during puberty in males could contribute to the sex‐specific differences in LD that may arise during development from child‐to‐adult. In rat skeletal muscles the findings are inconsistent. For example, in old rats with low LD‐M at baseline (M = muscle type; the main isoform in skeletal muscle), anabolic steroids increased the level of this enzyme, while in young rats with high level of LD‐M at baseline, anabolic steroids led to a decrease of this enzyme (Washington et al., 2014). Moreover, testosterone increased the activity of anaerobic alactic enzymes in rats, but the impact of testosterone on enzymes involved in lactate formation were not analyzed (Ramamani et al., 1999). In humans, we have not identified any studies on the effects of testosterone on glycolytic enzymes in skeletal muscle.

Secondly, fiber type composition may contribute to the sex‐related differences in LD in adults, because the relative type II fiber area is greater in males than in females (Simoneau & Bouchard, 1989). Together with the fact that LD activity is 3‐fold higher in type II than in type I fibers (Chi et al., 1983) this supports the idea that LD activity in a biopsy sample reflects the relative type II muscle fiber area. However, the existing correlations between LD and relative type II fiber area in adults are relatively weak (Esbjornsson et al., 1993; Simoneau et al., 1985).

Thirdly, the pattern of physical activity, such as type and intensity of exercise, may influence LD independently of both sex and relative fiber type area (Esbjornsson Liljedahl et al., 1996). Sex‐related differences in physical activity patterns may develop during the transition from child to adult. For instance, among children aged 5–18 years, the amount of vigorous physical activity decreased more in girls than in boys over this period (Corder et al., 2016).

### 4.2 Major findings—second hypothesis

The second hypothesis, that the fiber CSA is a determinant of glycolytic capacity in children, was also confirmed. The relative fiber type area may contribute to explain the variation in LD activity. However, the fiber CSA was a strong determinant of LD activity even after adjusting for relative fiber type area. The question arises as to why there is a relationship between the fiber CSA and LD activity and whether there is causality. Possible confounding factors could be body dimensions, age or the oxidative capacity of the muscle tissue.

An unexpected finding in the current study was that the oxidative enzyme CS also was a determinant of fiber CSA, through its inverse relationship with fiber CSA. This means that children with the smallest fiber CSA had the highest CS activity. Recent studies of mechanisms regulating the major isoform LD‐M show that activation of peroxisome proliferator‐activated receptor gamma co‐activator 1‐alpha (PGC‐1α) suppressed the expression of LD‐M in myocyte experiments (Summermatter et al., 2013). This suggests a coupling between the activation of oxidative pathways as indicated by PGC‐1α and the suppression of glycolytic markers such as LD‐M.

Furthermore, by using an interesting experimental model, it was shown that small muscle fibers have the highest maximal oxygen consumption and with increasing fiber size, a constraint of maximal oxygen consumption was being observed during unlimited oxygen supply (van Wessel et al., 2010). Oxidative metabolism in small fibers could therefore be favored by short diffusion distances between the muscle fibers and surrounding capillaries. Old data comparing mammals with a wide range of body mass show a positive correlation between glycolytic enzyme activity such as LD and body size and a negative correlation between oxidative enzyme activity such as CS and body size (Emmett & Hochachka, 1981). In growing rats, glycolytic enzymes increased and oxidative enzymes decreased until they reached early senescence (Singh & Kanungo, 1968).

To the best of our knowledge, there are no previous studies on the relationship between CSA and metabolic enzymes in children. Interestingly, however, Berg et al. (1986) reported that age was a positive determinant of LD activity in skeletal muscle and a negative determinant of oxidative enzyme activities such as CS and fumarase in a group of children aged 3–14 years. Since fiber CSA in children is related to age, these findings concord with the outcome of the present study.

### 4.3 Limitations

For ethical reasons and because of the children's thin muscle bellies, all measurements were based on a single biopsy sample per child, so we had to carefully select the most relevant analyses. Besides the morphological analyses, two enzymes were quantified from homogenates, representing oxidative and glycolytic metabolism. The selected enzymes are widely used in muscle research. CS was chosen because its activity is a strong marker for mitochondrial volume fraction and thus for muscle oxidative potential (Larsen et al., 2012; Reichmann et al., 1985). The main reason for choosing LD was that this enzyme is commonly used as a marker of glycolytic capacity in adult male and female skeletal muscle and demonstrates a consistent sex‐related difference (Esbjornsson et al., 1993; Essen‐Gustavsson & Borges, 1986; Green et al., 1984; Jaworowski et al., 2002; Simoneau et al., 1985; Simoneau & Bouchard, 1989). Therefore, we thought it was most logical to analyze LD activity in the children for the comparison with adult data. However, the choice of marker for glycolytic metabolism may not be critical as there is a coordinated regulation of glycolytic enzymes (Webster, 2003).

## 5 CONCLUSION

The typical adult pattern of higher skeletal muscle glycolytic capacity in males compared with females is not observed in children. Thus, the earlier known sex‐specific patterns in glycolytic capacity appear to develop during the transition from childhood to adulthood. Secondly, based on statistical analysis in the present study, muscle fiber cross‐sectional area is a determinant of both the glycolytic enzyme LD and the oxidative enzyme CS in children, regardless of sex. These data provide novel insight of muscle metabolic and morphological characteristics in boys and girls and of the development of these characteristics during the transition from childhood to adulthood, by the comparison with established sex‐specific differences in adults.

## AUTHOR CONTRIBUTIONS

Monica Dahlström, late Lennart Kaijser and Eva Jansson conceived and designed research; Monica Dahlström, Mona Esbjörnsson, and Jan Gierup and late Lennart Kaijser performed experiments; Mona Esbjörnsson and Barbara Norman performed morphological and enzyme analyzes; Mona Esbjörnsson analyzed data; Eva Jansson, Mona Esbjörnsson and Barbara Norman interpreted results of experiments; Mona Esbjörnsson and Barbara Norman prepared figures; Eva Jansson and Mona Esjörnsson drafted manuscript; Eva Jansson, Mona Esbjörnsson, Barbara Norman, edited and revised manuscript; All authors approved final version of manuscript.

## CONFLICTS OF INTEREST

None of the authors has any conflict of interest to disclose.

## ETHICS STATEMENT

We confirm that we have read the Journal's position on issues involved in ethical publication and affirm that this report is consistent with those guidelines.
